# Childhood Injuries in Singapore: Can Local Physicians and the Healthcare System Do More to Confront This Public Health Concern?

**DOI:** 10.3390/ijerph13070718

**Published:** 2016-07-16

**Authors:** Alvin Cong Wei Ong, Sher Guan Low, Farhad Fakhrudin Vasanwala

**Affiliations:** Department of Family Medicine, Sengkang Health, Alexandra Hospital, 378 Alexandra Road, Singapore 159964, Singapore; low.sher.guan@singhealth.com.sg (S.G.L.); farhad.fakhrudin.vasanwala@singhealth.com.sg (F.F.V.)

**Keywords:** childhood injuries, emergency visits, Singapore, preventable accidents, prevention strategies

## Abstract

Childhood injury is one of the leading causes of death globally. Singapore is no exception to this tragic fact, with childhood injuries accounting up to 37% of Emergency Department visits. Hence, it is important to understand the epidemiology and risk factors of childhood injuries locally. A search for relevant articles published from 1996–2016 was performed on PubMed, Cochrane Library and Google Scholar using keywords relating to childhood injury in Singapore. The epidemiology, mechanisms of injury, risk factors and recommended prevention strategies of unintentional childhood injuries were reviewed and described. Epidemiological studies have shown that childhood injury is a common, preventable and significant public health concern in Singapore. Home injuries and falls are responsible for majority of the injuries. Injuries related to childcare products, playground and road traffic accidents are also important causes. Healthcare professionals and legislators play an important role in raising awareness and reducing the incidence of childhood injuries in Singapore. For example, despite legislative requirements for many years, the low usage of child restraint seats in Singapore is worrisome. Thus, greater efforts in public health education in understanding childhood injuries, coupled with more research studies to evaluate the effectiveness and deficiencies of current prevention strategies will be necessary.

## 1. Introduction

Childhood injury is one of the leading causes of death globally [1,2,3,4], leading to about 40% of all child deaths [1,2,4]. According to the latest World Health Organization’s (WHO) data repository for 2015, injuries accounted for 364,824 deaths in infants and children aged between 0–4 years [5]. It is a growing public health problem that needs to be urgently tackled and looked into, both in developing as well as developed countries [2]. In the United States, almost 14% of emergency department visits in the population under 18 years of age are injury related [6]. Fatalities from childhood injuries are just the tip of the burgeoning iceberg, the morbidity implications of non-fatal childhood injuries contribute to significant healthcare and social burden as well as cost considerations. Injuries may result in physical, mental or psychological disabilities which adversely affect the quality of life of the casualties as well as their caregivers [2]. The physical and emotional care needs and burden may be enormous and difficult to quantify with the available statistical data. Utilising the concept of disability-adjusted life year (DALY) as a surrogate indicator, it is estimated by WHO that within the age group of 0–14 years, 1.2 million DALYs is lost in the higher income population, 19 million DALYs for the middle income and 28 million DALYs for the lower income population, respectively [2]. 

In Singapore, accidents, poisoning and violence were the top causes of hospitalization from 2012 to 2014 (8.3% to 8.4%) across all age groups [7]. Locally, it is estimated that between 10% and 37% of children’s emergency department visits are related to injuries [8,9]. According to the 2014 annual report on registration of birth and deaths, external causes of morbidity and mortality was the top cause of death in the age group from birth to 19 years old [10]. Prevention efforts will be important to ensure a safe environment for our children while decreasing the incidence and severity of childhood injuries. Many studies have attempted to propose effective interventions to prevent childhood injuries [11,12,13]. Successful interventional programs have also been described in the past [14]. Very often, the success and applicability of interventions have to be tailored to the profile of the population-at-risk. However, there is a lack of literature review done for the local population in Singapore. For such a significant public health problem, it is important to identify the evidence-based epidemiology, risk factors, prevention strategies and role of the various stakeholders in preventing childhood injuries. This narrative review attempts to summarize the available evidence from published literature relevant to the local settings. Through identification and evaluation of the local risk factor profile and areas of deficiencies in the unintentional childhood injury prevention, we hope to propose relevant prevention recommendations for to be considered for adoption both at the community as well as nation-wide level [15].

## 2. Materials and Methods

A search for relevant articles was performed on PubMed as well as the Cochrane Library. Using the PubMed Advanced Search Builder, the following search terms were used:

Search 1: (child OR children OR childhood OR infant OR toddler OR paediatric OR pediatric OR paediatrics OR pediatrics)
Search 2: (accident OR accidents OR trauma OR injury OR injuries)
Search 3: (Singapore OR “Singapore” [Mesh])

Publication dates: 1 January 1996 to 8 March 2016.The final search on PubMed performed on 8 March 2016, using the intersection of all the above 3 search results and publication dates restriction yielded 380 articles. The abstracts of all 380 articles were reviewed manually to assess for its relevance to unintentional childhood injury in Singapore. 29 articles were selected based on their relevance from their abstracts.

A further search on the Cochrane Library (using similar search terms) did not yield any relevant articles. A final search on Google Scholar (using similar search terms) for available locally published journals that were not indexed on PubMed, revealed an additional article. The full text of all 30 articles were obtained and reviewed for the purpose of this review. 

**Figure 1:**
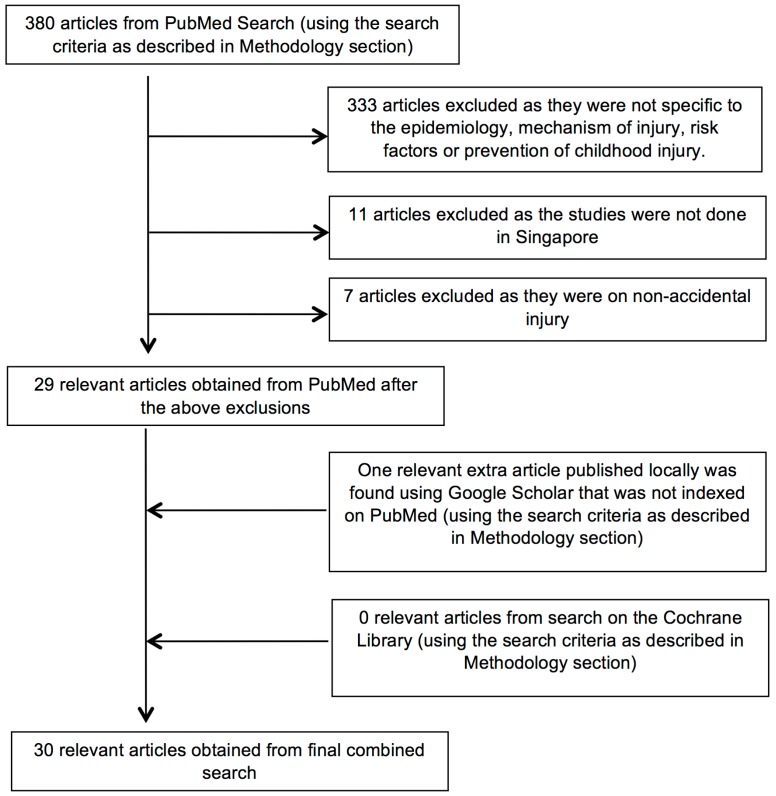
Details of the article selection process in the literature search.

The Strength of Recommendation Taxonomy (SORT) was used to grade the recommendations in preventing Childhood injuries in Singapore. The article selection process is summarized in Figure 1.

## 3. Results

Part A covers the epidemiology, mechanisms and risk factors of childhood injuries. The papers are grouped into review article, nation-wide and community studies; childhood injuries treated at emergency departments; childhood injuries related to specific devices or accessories; playground-related injuries; injuries related to drowning; and specific injuries.

Part B relates to the possible locally based prevention strategies in addressing issues mentioned in Part A. Part B is reviewed in the discussion section of this article.

For part A, a brief summary of the main papers is provided in Table 1.

### Part A—Epidemiology, Mechanisms and Risk Factors of Childhood Injuries

#### Review

A topic review on Childhood injuries in Singapore involving relevant publications from 1999 to 2009 reviewed the main findings and proposed clinical recommendations [30]. The key findings were: Home injuries and falls constituted the majority of the cases in both the community setting as well as at the emergency departments. Furniture, infant and child products were associated with the causation of injuries. Playground injuries and drowning accidents have also been described. Prevention strategies were discussed and clinical recommendations were proposed. The article concluded with three main recommendations, namely: cues with regards to childhood safety to be placed at locations with high incidence of childhood injuries; the usage of mass media to impart knowledge of home safety; as well as for healthcare providers to build upon their deficiencies in the area of childhood injuries prevention and the role they could play in helping parents and caregivers to decrease the incidence of unintentional childhood injuries [30].

A significant number of new publications and recommendations have arisen since the publication of the above-mentioned review. Thus, the current review attempts to comprehensively merge the key findings and recommendations from both current and past publications to provide a better insight into the current updated knowledge of childhood injuries in Singapore.

#### Nation-Wide and Community Studies

Studies have reported the local prevalence of childhood injuries at 7.7% to 19.5% [16,17,18]. Home injuries were the most common, accounting for up to 45%–91% of all injuries [16,17,18]. Falls was the main mechanism of injury in up to 92% of the cases [16,17,18].

A cross-sectional nationwide study published in 2005 involving 1293 households with 2322 children under the age of 15 years reported a 19.5% prevalence rate of children having one or more injuries in the past one year; with boys having a higher prevalence of injury (50.7%) compared to girls (48.6%). Most of the injuries (45%) happened at home, 32.2% of the injuries occurred outside the home and the remaining 22.8% at schools. It was noted that the majority (54.7%) of the home injuries occurred in the living room while playground and parks contributed to the most (44.9%) common place of injury occurring outside the building. The school field was reported to have the highest (78.9%) occurrence of injury in school. Regardless of the location of injury, falls was the main mechanism (76.1%–92.2%) of injury. A high prevalence of identified hazards was also noted in the study home. Loose items such as vases and decorations (44.7%), absence of non-slip bathroom mat (40.5%) and the presence of containers with water in the bathroom (31.6%) accounted for a higher proportion of identified hazards. There was an increasing incidence of injuries, which corresponded to the increasing number of hazards identified in the household [16].

From this similar study population, the authors also concluded that mothers with a higher educational background tend to have a better knowledge of childhood injury prevention. Mothers with tertiary education were three times more likely to have the correct knowledge on injury prevention and first aid compared to mothers with primary education or no education (Rate-Ratio 3.1, 95% CI 2.1–4.6). Though most caregivers were relatively well informed with regards to road safety; however, information on first aid skills, as well as on the knowledge and prevention regarding home injuries were found to be inadequate, and this issue needs to be tackled. Only a minority (38.5%) proportion of caregivers received information with regards to child safety through medical personnel, most caregivers do so via the media channels (64.7%) and from their family members (66.7%) [17].

Another descriptive analysis of unintentional infant injuries involving 405 infants aged one year old or below over a six month duration demonstrated that 7.7% of all attendances (in children aged one year or younger) at emergency departments, hospitals and primary healthcare facilities are related to infant injuries. Infants between 9 to 12 months old accounted for the majority (37%) of the cases compared to the other age groups. 91% of the injuries happened at home, with the bedroom being the most common (60.5%) place of occurrence. Almost 50% of the cases were associated with furnishing such as beds or bedding. The main mechanism of injury was due to falls (77%) and falls from beds or cots was the most common scenario. It was alarming that only 12 cases (3.9%) indicated having some sort of safety features for instance, safety barriers, non-slip mats, cot rails and seat belts. Head injuries were present in 63.6% of the cases. Serious head injuries accounted for 7% the head injuries. 17.9% of the study population required hospital admission [18].

#### Childhood Injuries Treated at Emergency Departments (ED)

Most of the injuries presented at the ED, involved injuries sustained at home (52.2%–56.4%) [9,19]. Falls were implicated in 49%–71.8% of the ED attendances [9,19,31]. A retrospective study involving 2517 children aged 12 years and younger presenting to an ED over one year duration noted that there was a high incidence of injuries sustained at home (56.4%) compared to other mechanisms of injury. Falls accounted for 66% of the injuries sustained at home. 62.7% of the all injuries involved the males. Home injuries tend to be more common in pre-school children (under the age of 5 years) (79.4%, *p* < 0.0001) compared with children aged between 6 and 12 years (41.0%). Pre-school children (under the age of 5 years) had a higher proportion of head injuries (*p* < 0.0001), foreign bodies injury (*p* = 0.0008), burns or scalds (*p* = 0.0009) and poisoning (*p* = 0.0331) compared to school-going children (aged 6–12 years). On the other hand, school-going children (aged 6–12 years) had a higher occurrence of injuries sustained at playgrounds (*p* < 0.0001), road accidents (*p* = 0.0002), sports (*p* < 0.0001) or at school (*p* < 0.0001) compared to pre-school children (under the age of 5 years). School-going children had a higher proportion of injuries involving their limbs (*p* < 0.0001), trunks as well as cases of multi-trauma (*p* < 0.0001) compared to pre-school children (under the age of 5 years). Road traffic accidents accounted for 7.4% of all injuries. It was surprising that only 6.1% of the children (aged 12 years and below) were placed in proper child restraint seats. 21.5% of all injuries (in children aged 12 years and below) were admitted to the hospital. Children aged 6–12 years had a higher likelihood of being admitted (*p* = 0.04) or referred for an outpatient follow-up with a specialist (*p* < 0.0001) compared to children aged below 5 years, whom are more likely to be discharged (*p* < 0.0001) [9].

A recently conducted prospective observational study involving 1049 children under the age of 16 years whom presented with head injuries to the ED published its findings in the British Medical Journal Open. Mortality rate of all attendances was 1.6%. The most common cause resulting in head injuries was due to falls (71.8%). 52.2% of falls (with head injuries) happened at home. In the sub-group analysis of children less than 2 years old, 84.7% of their injuries were falls-related and 67.9% of the injuries were at home. Furniture such as adult beds and floor surface were implicated in these injuries. It was found that with every metre increase in the height of fall, there was a significant higher likelihood of resulting in a severe outcome (Odds Ratio 1.4, 95% CI 1.3 to 1.6); this was found to be consistent amongst the individual severe outcomes of death (Odds Ratio 1.5, 95% CI 1.3 to 1.7) as well as the need for intubation (Odds Ratio 1.5, 95% CI 1.3 to 1.7). 11.7% of all head injuries were caused by motor vehicle crash. 75% of the children whom should have been either seated in child restraint car seats or have their helmets donned on while riding bicycles failed to do so. This is especially worrisome with the study demonstrating a higher likelihood (odds ratio 7.2, 95% CI 4.3 to 12.0) of a motor vehicle crash causing death, need for airway or neurosurgical intervention; as compared to falls-related head injury. In this study, a multivariate logistics regression analysis involving age, cause of injury and location of injury demonstrated motor vehicle crashes (*p* < 0.001), non-accidental trauma injury (*p* = 0.002) and injuries that occurred outside home (*p* = 0.007) as being statistically significant associating with a poor outcome [19].

Another case-control study comprising of children under the age of 16 years who was seen at the ED for head injury concluded that road traffic accident as the mechanism of injury in 44% of the head injuries with traumatic brain injury compared with road traffic accident being implied as the mechanism of injury in only 2% of those presenting with head injury but did not sustain any traumatic brain injury (*p* < 0.001). In children aged 2 years and below, road traffic accident was significantly associated with an increased incidence of traumatic brain injury compared to other mechanism of injury (*p* = 0.012). Falls was noted to be the main mechanism of injury (49%) but no significant association was found with the median height of the fall (*p* = 0.069). The investigators also concluded that four predictors of moderate to severe head injury was significant; namely: mechanism of road traffic accident (Adjusted OR 10.9, *p* < 0.001), difficult arousal (Adjusted OR 107.6, *p* < 0.001), vomiting (Adjusted OR 5.4, *p* = 0.003) and signs of base of skull fracture (Adjusted OR 44.1, *p* < 0.001) [31].

Shifting the focus to pedestrian injuries in children aged 16 years and below, most of the injuries were sustained by the males (60.5%) and between ages 7 to 12 years (50.2%). The bulk of the accident occurrence was on the roads (81.2%) and car parks (8.4%), peaking between 12 pm and 6 pm (51.3%). It was noted that most children were unaccompanied by an adult (67.8%). 6.5% of the pedestrian injuries resulted in major trauma. The authors also found that a positive history of being flung (*p* = 0.001) and loss of consciousness (*p* < 0.001) as being significantly associated with major trauma [20].

Most of the children aged below 16 years, whom presented with otorhinolaryngeal foreign bodies (FB) to the ED, were between the ages of 4 to 8 years (43.3%) followed by less than 4 years of age (39.4%). For presentations with ear FB, males (66.7%) formed the majority of the patients; with most of them between the ages of 4 to 8 years (43.3%), and toy parts being the most common object. For nasal FB, 56% were noted in the males with a highest proportion (56.1%) in children less than 4 years old. Beads, toy parts and organic matter (sweets, seeds, and peanuts) were of the highest incidence in nasal FB. Fish bone (81.1%) was most commonly found in throat FB. 4.2% of all presentations required hospital admission [21].

7.4% of all cases with toxic exposure presenting to EDs were paediatric cases and most were from unintentional exposure (91%) that occurred at home [32]. In a separate prospective study involving children aged 12 years and below whom were admitted to a paediatric hospital for unintentional ingestion, it was found that 60% were toddlers aged between 1 to 3 years. Most of the unintentional ingestions occurred during the day. With regards to the type of unintentional ingestions, it was found that medications had the highest incidence (49%) followed by household liquids (16%). One of the main reasons given by the caregivers for the child not being supervised was being pre-occupied with housework (23%). It was also noted that the majority of the medications was kept in cupboards that were not locked, with the remainder placed on tables, refrigerators, or simply left in bags either before or after consumption [22].

#### Childhood Injuries Related to Specific Devices or Accessories

In a retrospective review of stroller-related and pram-related injuries involving 248 injuries in children aged less than 6 years, most of the injuries were related to blunt trauma (97.6%) while 2.4% from a crushing injury. 11.7% of the injuries were related to use of strollers or prams on escalators or stairs. These injuries occurred in 79.4% of the cases, despite being in the presence and supervision of adults. The majority of the injuries happened at home (46.8%). Falling or tripping is the main mechanism of injury (89.1%). 27.8% of the injuries involved open wounds, fracture or dislocation in 6.9% of the cases and 0.8% of the injuries involved moderate to severe head injury. Procedural interventions were indicated in 20.1% of the injuries and 17.7% of the patients were admitted to the hospital. The authors noted that injuries requiring procedural intervention were significantly associated with older age (*p* < 0.001) compared with children who were younger. Furthermore, entrapment injuries were also significantly associated with the indication for intervention within the ED (*p* < 0.001) compared with other mechanisms of injury [23].

It was reported that 76.5% of children, who was treated for traumatic foot injury sustained on escalators, were wearing rubber clogs during the time of injury. Within this group, 69.2% had severe foot injuries due to entrapment, requiring hospitalization for emergency surgery. In these cases of severe foot injuries, it was noted that it occurred despite the presence of adult supervision for all of them, and not surprisingly, the rubber clogs were all badly crushed. Severe injuries that were implicated included open fractures and digital amputation. The study showed that out of the severe foot injuries which were escalator-related, most (75%) of them were linked to the wearing of rubber clogs. The authors postulated that the broad toe box design, which appeared oversized, giving an erroneous impression of the actual distance of the foot to the surroundings. Coupled with the soft malleable material of the footwear, it is easily crushed when it is trapped by the escalator mechanisms [33].

In a local study looking into injuries related with the use of Heelys™ in children, all of the cases with injuries did not use any form of safety equipment. 10% of the injuries required manipulation and surgical interventions. All the cases involved an average of 4 weeks duration of immobilization of the affected limb post injury. Their treatment cost average $193 per patient in 2004 [34].

Up to 90% of infants attending a primary healthcare clinic developmental assessment session were noted to be using infant walkers on a regular basis. Within this group, 12.5% sustained one or more injuries. A longer duration of walker usage per day (more than two hours a day) was associated with a higher incidence of injuries (*p* = 0.43). Moreover, 10.8% of those who used walkers demonstrated abnormal or questionable results when evaluated with a developmental screening tool (Singapore modified version of the Denver Developmental Screening Test), compared to 100% normal results in the group of infants who were not using any walkers [24].

The usage of sarong cradles can be hazardous as illustrated by the findings of a local study which showed that 6.5% of the children who visited the ED with head injuries were associated with the usage of sarong cradles. 26.3% of these injuries warranted hospital admission. Significant injuries like occipital fractures and extradural haematoma had been noted in the usage of sarong cradles in children. The authors concluded that there was a risk of severe head injury with the usage of sarong cradles [35].

A cross-sectional study evaluating the knowledge, attitudes and practices among caregivers towards toys safety showed deficiencies in the knowledge of toy safety and there exists a necessity for educational and regulatory efforts in our local population. It was noted that demographic factors of the caregivers such as gender (*p* = 0.495), education (*p* = 0.450), role as a caregiver (*p* = 0.559), working in a job that involved children (*p* = 0.840) were not associated with a significant difference in their level of knowledge towards toy safety. 92.5% admitted having bought a toy that was not recommended for the stipulated age group of the child. More than 90% of this group of respondents bought an inappropriate toy because they either believe in that the toy would benefit the child in an educational sense or they assumed that the child had attained the requisite developmental milestone to handle the toy. 87.1% of the respondents encountered toy-related incidents in their children, with incorrect methods on handling the toys (44.1%) and inadequate caregiver supervision (37.6%) given as the main reasons for such incidents [36].

#### Playground-Related Injuries

The mean age of playground-related injuries is around 6.8 to 7.5 years [25,26,27,28].There is a notable high incidence of monkey bars related injuries (49% to 66%) across the various studies [25,26,27]. 

Results from a cross-sectional study of 19,094 childhood injuries showed that playground-related injuries accounted for 8.5% of all injuries; with an increasing male predominance with increasing age groups (*p* = 0.006). The occurrence of most playground related injuries peaked in the time period between 1800 and 2100 h (37.6%), followed by 1500 to 1800 h (27.6%). It also occurred more frequently over the weekends and school holiday periods. The majority of these injuries were caused by falls (70.7%). The three most common equipment related to playground injuries were monkey bar or other playground climbing apparatus (52.1%), the slide (21.2%) and the swing (6.3%). 18.2% of all playground-related injuries involved upper limb fractures and 3% of the injuries involved lower limb fractures. 8.8% of all playground-related injuries required hospitalization. It was found that the risk of sustaining an upper limb fracture at a playground was highest amongst children who were between 6 to 10 years of age compared to the other age groups (*p* < 0.001). Falls arising from more than 1 m in height had a 4.1 times higher risk of an injury involving upper limb fractures (OR = 4.1, *p* < 0.001) compared to falls from less than 1m in height. Of note, children who were not accompanied by any caregivers were 1.4 times more likely to sustain upper limb fractures (*p* < 0.05) compared to children whom are accompanied by caregivers [25].

A retrospective review involving fractures of the extremities seen at a local children’s hospital correlated that 19.5% occurred in conjunction with the usage of playground equipment. Monkey bars accounted for most of the injuries in this group (66%) compared to other modes of injury. Supracondylar fractures (23.6%) were the most commonly sustained site of fracture injuries compared to other sites of fracture injuries. The proportion of monkey bars in the playgrounds and their level of usage in the study subjects were not reported in the study [26].

A more recent prospective study of playground related fractures involving the extremities that were seen at a children’s hospital noted forearm fractures (36%) and supracondylar fracture of the distal humerus (29%) were among the most common fractures that were sustained. Monkey bars were associated with 49% of the injuries, while injuries associated with slides accounted for 14%. Surgical intervention was indicated in 11% of the cases while hospitalization rate stands at 1.2%.The mean length of hospitalization was 1 day with a mean follow-up duration of 67.2 days. Treatment costs ranges from $247.50 to $3792.50. The proportion of monkey bars in the playgrounds and their level of usage in the study subjects were again not reported in this study [27].

The presence of any supervision was found to significantly correlate with a lower incidence of major fracture compared to injuries sustained in the absence of any supervision (*p* = 0.000). In those group of injuries sustained in the presence of supervision, it was found that supervision from parents (18.8%) or siblings (16.7%) resulted in a lower incidence of major fractures, compared to grandparents (27.3%) or domestic helpers (25.9%). However, only the presence of supervision from parents was statistically significant (*p* = 0.004). Interestingly, statistics reflected a trend of increased incidence in major fractures occurring in children with a Body-Mass Index (BMI) at either ends of the extreme (10 percentile or less and 95 percentile or more) (37.9%) compared to those with a BMI between 11 to 94 percentile (27.3%) (*p* = 0.074). The authors postulated that children who are heavier in weight may be less agile and may sustain a greater impact when they land from a fall in view of a heavier weight. However, they were unable to explain the relationship between fractures and a BMI at the low extremes [28].

#### Injuries Related to Drowning

The number of reported drowning victims in Singapore averaged 9.3 per year (range 2 to 12 per year) in the age group of 0 to 19 years [37]. In a retrospective study looking at the epidemiology of paediatric drowning and near-drowning locally, 38 cases were picked up and nine deaths were reported. The majority of the children were males. 52.6% of the accidents occurred in swimming pools. Within this group, 55% was within private home and condominium swimming pools. 21% of the accidents occurred in the sea. There was a higher mortality rate from accidents in the sea (62.5%) compared to accidents in the swimming pools (10%). The incidence peaked between 1400 and 1900 h as well as during weekends. 76.4% of the cases were unwitnessed by any caregivers. A vital finding that no safety features (such as life guards, floatation devices, fencing, etc.) was noted in 47.4% of the cases. The data also showed a 100% survival rate for near-drowning accidents in pools which had a lifeguard around. The authors made a special mention of bathtubs, jacuzzi and ponds as a hazard to toddlers and pre-school children [29].

#### Specific Injuries

Sports-related injury is important in older children as they become more active and participates in different kind of sports. Injuries related to various overuse mechanisms are not uncommon. In a retrospective study conducted for 506 cases of sports related overuse injuries in children, the knee joint was injured most commonly (66.9%) compared to the other sites of injuries. There was a male predominance (73%) in the study population. The mean age at diagnosis of overuse sports-related injuries tend to be older in males (11.7 years of age) compared to female (10.8 years of age) (*p* = 0.001). Osgood-Schlatter disease (OSD) was noted to be the most common diagnosis. Majority of the injuries were related to running and ball-type activities [38].

In a study involving children aged between 2 to 14 years whom were seen at a paediatric hospital, tripping and falling on an outstretched hand while running accounted for most of the radial neck fractures sustained by children (40.7%); while falling from monkey bars constituted the second highest incidence (mechanism of injury) at 10.2%. Injuries with a higher fracture grade was associated with a worse prognosis (*p* = 0.001) and more invasive interventions (*p* = 0.001) compared to injuries of a lower fracture grade. There is a higher tendency of more severe fractures (*p* = 0.04) and poorer outcomes (*p* = 0.007) for older children compared to younger children. It was suggested that a greater magnitude of energy may be involved in injuries involving children who are older. The bones of younger children are also more cartilaginous and offer a better cushioning effect to absorb the energy from a trauma, resulting in a lesser severity of fractures. Moreover, the bone structure of younger children has a greater remodelling potential to facilitate a better prognosis [39]. 

In a retrospective review of 181 children with hand fractures, there was a steep increase in incidence of hand fractures from 11 years of age with peaks at 14 years and 15 years. A male predominance was noted. The two most common fractures involved the proximal phalanx (49%) and the fifth ray (40%) of the hand. School was the most common place where hand fractures are sustained (44%), surpassing playgrounds and sports venues (32%). For the age group 0–5 years, home injuries (72.7%) were the commonest. With regards to the mechanism of injury, crushing injuries were predominant for children aged 0 to 5 years; whereas sports (39%) and falls (28%) were predominant in the older children [40].

In another retrospective analysis of 202 children with fingertips injuries, boys (60%) as well as children aged two years (14%) were noted to have a higher risk of sustaining the injury. Overall, crush injuries emerged as the commonest injury mechanism (77%) and door injuries was the most common cause (87%) of crush injuries. Inpatient surgery was performed in 58% of the patients and outpatient surgery for 20% of them. The left ring finger was injured in most of the cases followed by the left middle finger [41].

## 4. Discussion

### 4.1. Part B—Prevention Strategies and Recommendations from the Studies Reviewed

The prevention strategies and recommendations from the studies that were reviewed are systematically discussed under the preventive framework categories of “Education strategies”, “Engineering Strategies” and “Enforcement strategies”.

#### 4.1.1 Home Injuries

##### Education Strategies

Home injuries are very common in children [9,16,18,19,22,23,30,32,40]. There may be a wrong perception among caregivers that the home environment is a safe place for their children. It is important to raise awareness that common hazards such as sharp edges or corners of household furniture, cluttered furniture, dangling electrical cords are important causes of injury and should be addressed [16,19,30]. Sharp equipment such as knives and scissors should be kept out of the child’s reach [16,30]. Education and interventional programs to raise caregivers’ awareness and to help them to understand child safety and preventable injury strategies in ensuring a child-safe environment will be essential [16]. More emphasis on first aid information and fall prevention strategies should be provided for as well [17,18]. The role of health education and provision of injury prevention advice can be promoted by medical and nursing professionals during antenatal or postnatal visits, as well as immunization and developmental assessment sessions [17,18]. More publicized education at dissuading local families from nursing infants or children in adult beds and to encourage the use of proper cots will be needed [9]. Caregivers should be cautioned to keep beads, small or broken off toy parts, small objects like seeds, peanuts, pencil lead, cotton buds or button batteries, away from the children’s reach [21]. They should also be careful to look out for the presence of any fish bones that may be in the food given to the child [21].

Regular education to both caregivers and children on poison prevention and proper storage of drugs and chemicals at home to ensure that they are inaccessible to children will be important [22,32]. They are best stored in cupboards that are securely locked and away from the children. Medications and hazardous solutions should not be kept in beverage bottles which may be implicated in unintentional ingestion of their contents. Caregivers should also ensure that the correct medication is being served to the child by prior counter-checking of the label.

Information on home-safety can also be disseminated in the form of pamphlets and posters through primary care clinics and child-care facilities. Such information can also be disseminated through organised talks given by trained professionals [22].

##### Engineering Strategies

Windows grills and locks should be installed at home [9,16]. The usage of safety gates near stairs, non-slip mats or tiles in the bathrooms, proper latches, door stoppers or self-closing hinges on home doors will help to prevent unintentional home injuries [9,41]. Caregivers should be advised not to use tablecloths, but to use place mats instead, to prevent the incidence of unintentional scalds [16].

The manufacturing of drugs in child-proof containers, opaque blister packs or strips may help in reducing the incidence of childhood unintentional ingestions [22].

An age-specific safety checklist may be considered for inclusion into the child’s health booklet [18].

#### 4.1.2. Childcare Products and Footwear

##### Education Strategies

The dangers and potential morbidity caused by the use of sarong cradles should be publicised and have its usage discouraged [9,18,35]. If caregivers insist on using sarong cradles, dedicated supervision by a responsible adult should be ensured. They should ensure the usage of a proper sarong length and make routine inspections for any potential defects. 

Caregivers should always ensure that the seatbelts be fastened when children are seated in high-chairs [9].

Prior to using strollers or prams, safety checks with appropriate adjustments to ensure a stable and well-balanced structure as well as the absence of any exposed joints or hinges should be performed. The safety harness should be promptly secured once the child is placed within the device. Children must be constantly supervised. They should not be leaning out of, or be standing in strollers or prams. Parents should be aware of the following factors in the selection of strollers or prams, namely: age and size-appropriateness of the pram or stroller, the presence of efficient brakes, locks and safety harnesses, as well as if the mandatory safety features are in compliance with international safety standards or certifications [23]. 

Education on the safety precautions in the usage of Heelys™ should be emphasized. Children should be encouraged to don safety gear and be under close supervision of adults when using Heelys™ to avoid sustaining preventable injuries [34].

Appropriate education should be given to parents with regards to the potential dangers as well possible influence of on the motor development of children who utilize infant walkers. The safer alternative of using a crib or playpen instead of an infant walker should be make known to the parents [18,24].

Parents should routinely inspect to ensure that their children’s toys are free from any broken components, chipped paint, damage and potential hazards. If any of these are present, they should be replaced or be repaired right away. Parents should supervise their children during playtime and ensure that toys are being handled correctly. It is also important to educate them that the age recommendation of toys are not determined by its level of difficulty for the specified age group, rather, it is due to the potential hazards that the toy may pose to the child such as the presence of small parts. Thus, parents should be advised to adhere to the recommended age group of each toy in their selection for their children. This will reduce the incidence of unintentional injuries that are related to toys [36].

##### Engineering Strategies

An appropriate protective material may be used around the area of the sarong cradle to prevent unintentional injuries [35]. 

Special labels and cues sited near common injury prone locations (of prams and strollers) such as stairways and escalators should be implemented. To discourage the hazards of prams and strollers being wheeled onto escalators, barricades may be fixed at their entrances and exits [23].

Escalators with essential safety features such as brushing and step safety side plates, coupled with regular maintenance schedules and regular lubrication of their side panels will help in decreasing the incidence of escalator related injuries. The recommendations in preventing rubber clogs related foot injuries while travelling on escalators are summarized in a separate table at the end of the article. 

##### Enforcement Strategies

Some studies advocated the use of legislation to disallow usage of sarong cradles [18].

#### 4.1.3. Playground-Injuries

##### Education Strategies

Advice on the safe usage of playground equipment should be displayed prominently at both public and private playgrounds to increase awareness of their appropriate use among both caregivers and children [25]. The mean age group of many studies were very well correlated [25,26,27,28], parents should be extra cautious in supervision of the children who are around the age group of 6 to 8 years when they are playing in a playground. In addition to providing proper supervision of children playing in the playgrounds, caregivers should also try to maintain the child’s BMI within the recommended limits, as this may possibly decrease the incidence of severe fractures [28].

##### Engineering Strategies

A conscientious review of the heights of common playground equipment to ensure that they are age-appropriate is needed to prevent the incidence of falls and the resultant injuries [25]. Monkey bars should be substituted with safer equipment [9,25,26,27]. It has been suggested to restrict the maximum height of any hanging equipment to 1500 mm [9,26].

#### 4.1.4. Transport Related (Child Restraint, Helmets, Cycling, Pedestrian)

##### Education Strategies

More publicity efforts should be made to boost the awareness with regards to the updated legislation in Singapore with regards to the obligatory usage of appropriate child restraints or booster seats in cars for anyone below the height of 1.35 m [9,17,18,19,42]. Another study advocated the introduction of child safety programs at the ED [19].

Educational efforts to publicize safe cycling practices and proper utilization of safety equipment while cycling should be emphasized. Awareness of the mandatory need for children to wear helmets while riding a bicycle on Singapore roadways should be promoted [19]. 

Most of the childhood pedestrian injuries involving primary school children who were unaccompanied on their way back home [20]. Parents should be advised to disallow young children to manoeuvre roads or car parks alone but to ensure that they are properly supervised. 

##### Engineering Strategies

The usage of helmets while riding on a bicycle has been shown to significantly reduce the incidence of head injury and facial injury [43]. Wearing of proper footwear for cycling also helps in the prevention of associated injuries [9].

##### Enforcement Strategies

It is noted that the usage of age-appropriate child seats is relatively low despite knowledge of its requirement in a majority [9,17,18,19]. Thus, a stricter enforcement of this law is glaringly necessary [9]. One study even suggested the need for a revision of this law with the implementation of a “civilian volunteer reporting scheme”, akin to having whistleblowers, in an attempt to increase compliance as well as decrease preventable injuries for children involved in motor vehicle accidents [9].

Mandatory use of helmets while riding bicycles at all times should be reiterated [9]. 

#### 4.1.5. Drowning and Near Drowning

##### Education Strategies

It has been suggested that a multi-pronged approach that age-appropriate is necessary in the prevention of drowning [29].

Caregivers should be advised to avoid having water-filled pails at home or to ensure that children do not have access to these pails or ponds at home [9,16,29]. An adult should always be present when bathing infants or toddlers [29]. Supervision by an adult when children are at swimming pools, seas or any water features, is likely to reduce the incidence of drowning accidents [29,37]. The acquiring of swimming skills and access to aquatic safety education should be highlighted among all children [37].

##### Engineering Strategies

Essential safety features such as lifeguards or swimming instructors, an enclosed four-sided fencing, self-closing access points, pool alarms and rigid covers, should be made available at swimming pools [29,37]. At the sea-side, essential safety features include lifeguards, warning boards and floatation devices, clear delineation of swimming areas with buoys and markers as safety features, rings buoys, poles and conspicuous signs [29,37]. Aesthetic features at pools may be hazardous, and should be minimized [29].

##### Enforcement Strategies

The relevant authorities should ensure that as many essential features as discussed above to be made available at swimming pools as well as sea-sides [29,37].

One study proposed the mandatory presence of lifeguards prior to any permitted swimming activities in public and condominium swimming pools and designated seaside-swimming zones at specific hours [29]. Children should only be allowed in designated seaside-swimming zones with lifeguards who are on duty [29]. Formal legislation of the need for lifeguards and pool fencing should be considered [9,29,37].

#### 4.1.6. Healthcare Professionals

##### Education Strategies

The role of health education and provision of injury prevention advice can be promoted by medical and nursing professionals during antenatal or postnatal visits, as well as immunization and developmental assessment sessions [17,18].

Physicians should also be familiar with evidenced-based predictors in children who sustained head injury so as to promptly recognize and initiate appropriate referral for closer monitoring and interventions of the significant injuries [31].

Physicians working in the ED setting should pay particular attention to childhood head injuries which are associated with a road traffic accident, difficult arousal, base of skull fracture, vomiting, and a positive history of being flung and loss of consciousness [31].

Physicians should also be aware of the possible red flags to watch out for when managing a child who presents with a possible otorhinolaryngeal foreign body. A good knowledge of common presentations such as local pain, appropriate history taking as well as the local epidemiology will be vital in the proper management of such commonly encountered cases [21].

Physicians should be familiar of developmental differences between the different age groups of athletes to enable them to appropriately manage sports related injuries as well as the expectations from parents and coaches. Physicians should also attempt to advance their knowledge and experience of the hazards and safety aspects of organized sports that is characteristic to the individual sport [38]. Having a good knowledge of the common areas of hand phalangeal fractures that are specific to different age groups will be helpful in the diagnostic process. Particular attention should be given to exclude phalangeal fractures in children who are younger and fractures of the metacarpal bones in teenagers in the absence of a good history [40].

In the paediatric inpatient setting, providing family and caregiver education of fall prevention strategies helps to increasing the caregiver’s awareness and leads to a decreased incidence in falls. A successful outcome is also dependent on good communication of the importance of fall prevention to both the staff as well as caregivers [44].

#### 4.1.7. Establishment of a Surveillance Database; Public Education and Legislation

##### Education Strategies

Most studies concluded that substantial efforts are needed in the foray of public education and intervention programs to increase awareness, correct misconceptions, boost safety compliance in reducing the incidence of preventable childhood injuries [9,16,17,18,19,20,21,22,23,24,25,26,27,28,29,30,31,32,33,34,35,36,37,38,39,40,41,43,44]. Such outreach measures may be effectively disseminated through the mass media, hospital and community resources [9,17,19].

##### Engineering Strategies

Some studies advocated the establishment of a robust injury surveillance database for epidemiological analysis and enabling targeted future initiatives for safety campaigns and injury prevention [9,25]. Such database may be incorporated on a nation-wide basis with inputs from primary healthcare providers [9].

##### Enforcement Strategies

Legislation and enforcement to ensure compliance with the usage of child-safe devices and safety regulations as well disallowing the usage of specific infant care products which are significantly related to injury causation, should be deliberated on [9,18,29,37].

### 4.2. Appraisal and Grading of the Recommendations on Prevention of Childhood Injuries in Singapore

In this review, we attempted to summarise the available key findings and evidence-based recommendations that are specific and relevant to our local population in the area of childhood injuries. Reviews and recommendations related to the incidence and prevention of global childhood injuries may not be entirely applicable to any country’s local population. Thus it is important to have an updated review to provide knowledge of the current landscape of childhood injuries in Singapore, the deficiencies that need to be looked into, as well as effective recommendations that can help to tackle this important yet often preventable public health problem. 

Childhood injuries is common yet preventable. The Strength of Recommendation Taxonomy (SORT) is adopted to rate the strength of the proposed recommendations [45]. SORT addresses the quality, quantity and consistency of the appraised evidence and does not require the user to have any formal training to be able to understand it [45]. Thus, SORT is adopted for the purpose of this review. (Please refer to Table 2).

Apart from the burden of significant healthcare cost involved with childhood injuries [27,34], the morbidity, psychosocial and emotional implications on both the child and their caregivers are very often underestimated and overlooked [46]. For instance, traumatic brain injury in children has been shown to result in numerous impairments and disabilities [47]. This not only translates to a guarded quality of life for the children but also the enormous emotional and financial burdens on their caregivers as well as community resources [47]. From a philosophical point of view, a child has the rights to be in a safe and secure environment. Being dependent minors, they have to rely on parents, caregivers and stakeholders involved in policy making to create such an environment for them to live, play, nurture and grow in. It is thus our responsibility, as parents or caregivers to do our utmost best in curtailing the known hazards in their environment and accede to them their fair rights. Prevention is always superior to cure [46]. Any healthcare system may boost of the finest management of childhood injuries, but we believe that the best that we can give to our children is to take an active and deliberate approach to prevent such injuries in the first place. 

The vital risk factors and predisposing causes of childhood injuries in Singapore has been detailed and discussed in the earlier sections of this review. The subsequent important agenda will be to disseminate this knowledge and to build up awareness among parents, caregivers, teachers, health professionals, facilities administrators, legislators and among the children themselves. 

The high incidence of home injuries and mechanism of falls within homes warrants a review of common hazards in that may be present in households with children. Perhaps a checklist of common household hazards may be formulated and distributed to all families with young children. The dos and don’ts on how to childproof a safe home environment should be shared on various platforms such as the mass media, social media as well as at visits with health professionals and dedicated health education seminars. Special focus should be given to young parents and first time parents. 

The reported low compliance to the usage of appropriate child restraint seats [9,17,18,19] is of great concern, especially in light of a significantly high likelihood of a motor vehicle crash causing death and poor outcome in children [19]. The fact that road traffic accidents being significantly associated with moderate to severe head injury and traumatic head injury adds urgency to this issue [31]. The authorities play an important role in enforcement of this law. More importantly, the incongruence of the reported high awareness of the child restraint law and its indication (to prevent injury from accidents) among caregivers [17] and yet displaying the poor compliance towards it [9,18,19], remains unexplained. More studies are needed to understand the underlying reasons and to tackle the root issues beyond legislations. 

The presence of dedicated adult supervision especially for infants, toddlers and young children is important in the prevention of unintentional childhood injuries. They should be supervised closely, especially when they are at play, swimming, crossing the roads or coming into contact with small objects, childcare products and household items. In particular, caregivers should ensure appropriate usage of playground equipment is adhered to and refrain from letting their children to play with devices, which may be inappropriate for their age group. 

Safe and proper usage of childcare products and devices must be ensured at all times. Perhaps, the regulatory authority can consider implementing a safety rating system for strollers and prams based on international safety standards and certifications. Stroller and prams are widely used locally and the association with childhood injuries has been discussed above. With a simple and standardized safety rating system, it may help parents to understand more easily and select relevant safety features and designs with ease. Special precautions specifying the common injuries that may be associated with certain child-related accessories or devices such as rubber clogs, infant walkers and sarong cradles should be mandatorily labelled on these products so that parents can make an informed decision prior to any purchase as well as to be aware to look out for the potential occurrence of such injuries when using them. 

Parents should be encouraged to be involved in the activities and toys that their children are engaged in. This not only helps in ensuring the children are handling their play in the correct and safe manner, but also helps them to understand the potential risks that certain activities and devices may pose to the children.

There is a need for more updated studies involving playground related injuries in view of the changing landscape of the local playground environment over the years. Most of the available studies reported using data gathered more than 9 years ago. Newer studies to better reflect the current epidemiology of playground related injuries, will help facilitate a more relevant and targeted approach to reduce the incidence of such injuries. 

Healthcare professionals should poise themselves as advocates of health promotion and health education to both caregivers and their children [5]. In particular, family physicians and nurses in the primary healthcare sector and ED play an important role in opportunistic health promotion and education as they are likely to have more points of contact and interactions with the caregivers and the children. 

Schools should encourage children to take up basic swimming classes and the government can consider to subsidise the costs of acquiring basic swimming skills which can be potentially life-saving in any near drowning accidents. A dedicated committee should look into the adequacy of essential safety features in public, private and designated swimming areas in the sea and propose the necessary recommendations to determine if more stringent legislation or regulations are required to be imposed. Similar recommendations should be feed backed to the Buildings and Construction Authority, which is responsible for regulating Singapore’s building and construction industry, as well as to Housing and Development Board, which is responsible for regulating public housing and facilities in Singapore. The lack of windows grills in homes and safety features in pools may warrant the relevant legislation and regulations to be introduced to curb the incidence of related childhood injuries. 

Ultimately, it comes down to delicate balance of the flexibility versus how stringent rules and regulations should be imposed. As much as legislation may compel certain changes to be made, each of us still have a personal responsibility towards contributing to a child-safe environment. Laws can never take the place of a good system of public education. We also would not wish our children to be living in an artificial “safe bubble”. A systems approach over an individually-focused health education, is needed for effective prevention [46].

The results from local studies are comparable to the findings of the global population. In the report released by the World Health Organisation (WHO), unintentional injuries are implicated in 90% of the 950,000 fatalities in those aged below 18 years annually [2]. The main reported causes of childhood deaths were road traffic accidents, drowning, burns, falls and poisoning [2]. The global report also identified falls contributing to the majority of non-fatal childhood injuries [2], similar to the findings from the local studies done in Singapore. The findings from the report shared many areas of similarities with the findings in the Singapore population. For instance, it was reported that several studies done in different countries noted falls as a significant cause of playground related injuries; they also noted that reducing the height of playground equipment leads to a reduced severity of playground related injuries [2]. The WHO report also concluded 5 key approaches to tackle childhood injuries; namely, legislation, product modification, environment modification, education and skills development and emergency medical care [2]. These are largely similar to findings and recommendations as concluded in this current review of local childhood injuries in Singapore. Therefore, the results and recommendations from this review could possibly be applied to the populations in the other countries which share similar demographics as Singapore. 

Focusing on the epidemiological data of childhood injury in the Association of Southeast Asian Nations (ASEAN) countries, which are geographically closest to Singapore; childhood injuries have been shown to be a significant leading cause of death in children and adolescents aged 0 to 19 years in 2013. In Thailand, road injuries were the top cause of death while drowning ranked as the fourth more common cause. In Malaysia, road injuries accounted for the second most common cause with drowning in sixth place and foreign bodies in eleventh place. In Vietnam, road injuries ranked as the fifth most common cause with drowning in the fourth place. In Indonesia, road injuries ranked seventh and drowning ranked sixth. In Myanmar, road injuries ranked eleventh and drowning ranked eighth. In the Philippines, road injuries were the twelfth most common cause while drowning ranked tenth. Turning our attention to Japan, which is comparable to Singapore in terms of being a developed Asian country, road injuries were the third most common cause of death in the same age group (0–19 years) while drowning came in at a close seventh commonest cause [15]. In Hong Kong which has a population census close to Singapore, childhood injury is a major public health problem as well with injury and poisoning being one of the leading causes of death in children aged 1 to 14 years [48]. Falls and home injuries were significant causes of childhood injuries in Hong Kong [48,49,50]. External causes of death accounted for 21.0% of mortality between the ages 0 to 19 years between 2001 and 2009 [50]. These data closely parallels the findings of our study and support the notion that childhood injuries are a significant public health issue in countries which are geographically close to Singapore; as well as an Asian country like Japan and Hong Kong which have a similar highly developed economy and advanced technological infrastructure as Singapore. However, these countries do not share similar proportions of the multi-ethnic population make-up in Singapore, thus it will be still important for this review to summarise the epidemiological evidence of childhood injuries in Singapore.

The strengths of this review include being a comprehensive coverage of available literature relevant to the local population and an attempt to collate and grade varied evidenced-based recommendations from many studies. 

The limitations of this review include it being a narrative review; a systematic review or meta-analysis will be superior. Most of the studies included in this review are retrospective studies and cross-sectional studies, more high level evidence studies in the form of cohort studies or randomized controlled trials would be preferred. The age and injury definitions different among the various studies, this makes it difficult to compare results across the studies. The latest comprehensive nationwide study was done in 2005, the presence of a more recently conducted one will better reflect the updated epidemiology and profile of childhood injuries in Singapore. There was also a lack of statistical values provided in much of the findings across the different studies, thus it was difficult to determine the significance of some of the results. Moreover, in some of the studies, there was a lack of information on the exposure to risk factors (e.g., exposure to monkey bars in playground-related injuries), thus there is a need to consider such potential confounding factors that might affect the implied risk factors in such studies. Furthermore, most of the studies looked into the epidemiology, risk factors and outcomes of childhood injuries; there is a lack of data that evaluated the efficacy of preventive interventions. Thus most of the preventive recommendations were only accorded Grade C on the SORT grading scale. More dedicated local research to evaluate the efficacy of childhood injury prevention strategies should be done to provide stronger evidence-based recommendations.

## 5. Conclusions

In conclusion, childhood injury is a common, preventable and significant public health concern in Singapore. Home injuries and falls are responsible for majority of the injuries. Injuries related to childcare products or devices, playground, drowning as well as road traffic accidents are important causes as well. Healthcare professionals, various advocates of public education and legislators play an important role in raising awareness and reducing the incidence of childhood injuries in Singapore. Several injury prevention approaches has been summarized and proposed. Greater efforts in public health education in understanding childhood injuries, coupled with more research studies to evaluate the effectiveness and deficiencies of current prevention strategies will be instrumental to address this area of public health concern.

### 5.1. What Is Already Known about This Topic

Childhood injury is one of the leading cause of death globally, including Singapore.

Home injuries and falls accounts for majority of the injuries.

### 5.2. What This Study Adds

Updated epidemiological information, risk factors and recommended prevention strategies are discussed in this review. In particular, updates on pram or stroller related injuries, head injuries, motor vehicle related injuries, pedestrian injuries, drowning and near-drowning, playground-related injuries, knowledge of toy safety; and their causation with childhood injuries are presented and discussed.

## Figures and Tables

**Table 1:** Summary of Main Papers on Childhood Injuries in Singapore.

Reference	Study Population	Settings	Main Results	Key Recommendations
**Nationwide and Community Studies**				
Thein et al. 2005 [16]	1293 households 2322 children. under 15 years of age	Cross-sectional nationwide community. Survey of households.	Prevalence of injury: 19.5%	◆ Need to increase awareness of the importance of home injuries through education and intervention programs. ◆ Reduce home hazards to create safe environment.
			Location of injury	
			▪ Home: 45% ▪ School: 22.8% ▪ Outside of Building: 32.2%	
			Location of home injury	
			▪ Living room: 54.7% ▪ Kitchen: 17.7% ▪ Bedroom: 13.8% ▪ Bathroom 9.5%	
			Household hazards	
			▪ Crowded or cluttered furniture: 24.5% ▪ Furniture with sharp edge: 25% ▪ Loose items: 44.7% ▪ Water in containers: 31.6%	
			Increasing incidence of injuries, which corresponded to the increasing number of hazards identified in the household.	
Thein et al. 2005 [17]	1293 households 2322 children under 15 years of age.	Cross-sectional nationwide community survey of households.	Main caregiver	◆ Necessity for education on home safety and first aid. ◆ Education through the media as one of the most effective avenues. ◆ Role of healthcare professionals in opportunistic health education.
			▪ Mother: 68.5% ▪ Grandmother: 13.4% ▪ Maid: 9.9%	
			Information source on child safety	
			▪ Advice of parents and relatives: 66.7% ▪ Programs on child safety: 64.7% ▪ Health personnel: 38.5%	
			Education level is a clear predictor of the mother’s appropriate knowledge and practice on the prevention of childhood injury.	
			Mothers with tertiary education were three times more likely to have the correct knowledge on injury prevention and first aid compared to mothers with primary education or no education (Rate-Ratio 3.1, 95% CI 2.1–4.6).	
Snodgrass et al. 2006 [18]	405 infants aged 1 year old or younger, with unintentional injury	3 ED, 2 polyclinics (primary care centres), HSA.	Infant injuries: 7.7% of total attendances.	◆ Nurse infants in baby cots instead of adult beds to prevent falls-related injuries at home. ◆ Injury prevention counselling should be made a priority to be provided for caregivers of infants. ◆ Age-specific safety checklist should be implemented in child’s health booklet. ◆ Health education on child safety should be provided at antenatal and postnatal visits.
			Age range of study population	
			▪ 0 to <3 months: 6.9% ▪ 3 to <6 months: 30.4% ▪ 6 to <9 months: 25.7% ▪ 9 to 12 months: 37%	
			Home injuries: 91%	
			Falls-related injuries: 77%	
			Main locations of home injuries	
			▪ Bedroom: 60.5% ▪ Living room: 18.5% ▪ Kitchen: 4.4%	
			Objects involved in injury	
			▪ Furnishings (bed/chair): 49.9% ▪ Infant/child products: 19.5% ▪ Person/plant/animals: 12.3%	
			17.9% required hospital admission.	
			Lack of safety features (e.g., non-slip mats, safety barriers, cot rails and seat belts) in 96.1% of fall-related injuries.	
**Childhood Injuries treated at Emergency Departments (ED)**				
Ong et al. 2003 [9]	2517 Children aged 12 year and below presenting with trauma.	ED of a tertiary children’s hospital.	Mechanism of injury	◆ Legislation on window grills to prevent falls-related injuries, and child restraint seats to prevent road traffic accident related injuries. ◆ Usage of proper baby cots to prevent falls-related injuries at home. ◆ Ensure fastening of seat belt on high chair and usage of non-slip mats to prevent falls-related injuries. ◆ Safety gates to prevent falls from stairs. ◆ Usage of door latches to prevent injuries from slamming doors. ◆ Age appropriate toys to prevent small parts that predispose to foreign bodies injuries. ◆ Do not leave pails of water at home to prevent drowning/near-drowning accidents. ◆ Need for a national injury surveillance database.
			▪ Home: 56.4% ▪ Sports: 8.2% ▪ Road traffic accidents: 7.4% ▪ Playground: 7.4%	
			Hospital admission rate of all injuries: 21.5% 42.5% of road traffic injuries caused by car collisions.	
			Cause of home injuries	
			▪ Falls: 66% ▪ Mechanical injuries: 14.3% ▪ Slamming door injuries: 6.1% ▪ Foreign bodies: 4.4% ▪ Drowning/near-drowning: 0.4%	
			Home injuries	
			▪ In pre-school children (under the age of 5 years): 79.4% ▪ In children aged between 6 and 12 years: (41.0%). ▪ *p* < 0.0001	
			Pre-school children (under the age of 5 years) as compared to school-going children (aged 6–12 years), had a higher proportion of	
			▪ head injuries (*p* < 0.0001) ▪ foreign bodies injury (*p* = 0.0008) ▪ burns or scalds (*p* = 0.0009) ▪ poisoning (*p* = 0.0331)	
			School-going children (aged 6–12 years) as compared to pre-school children (under the age of 5 years), had a higher occurrence of injuries sustained at:	
			▪ playgrounds (*p* < 0.0001) ▪ road accidents (*p* = 0.0002) ▪ sports (*p* < 0.0001) ▪ school (*p* < 0.0001)	
			Children aged 6–12 years had a higher likelihood of being admitted (*p* = 0.04) or referred for an outpatient follow-up with a specialist (*p* < 0.0001) compared to children aged below 5 years, whom are more likely to be discharged (*p* < 0.0001)	
Chong at al. 2016 [19]	1049 Children aged less than 16 years. Admitted for head injury.	ED of 2 tertiary children’s hospital.	Mechanisms of injury	◆ Initiation of child safety programs at ED. ◆ Prompt recognition of abuse cases and management. ◆ Age-specific education of injury prevention. ◆ Usage of helmets and child restraint seats.
			▪ Falls-related: 71.8% ▪ Motor vehicle crash (MVC): 11.7% ▪ Sports: 6.1% ▪ Non-accidental trauma (NAT): 1.6%	
			Percentage of falls-related injuries at home: 52.2%	
			With every metre increase in the height of fall:	
			▪ A significant higher likelihood of resulting in a severe outcome (Odds Ratio 1.4, 95% CI 1.3 to 1.6) ▪ This was found to be consistent amongst the individual severe outcomes of death (Odds Ratio 1.5, 95% CI 1.3 to 1.7) as well as the need for intubation (Odds Ratio 1.5, 95% CI 1.3 to 1.7).	
			75% of road users not using helmets or restraints.	
			Odds ratio of causing death, need for airway or neurosurgical intervention, compared to falls-related head injury:	
			- MVC: 7.2 (odds ratio 7.2, 95% CI 4.3 to 12.0) - NAT: (odds ratio 5.8, 95% CI 1.8 to 18.6)	
			Multivariate logistics regression analysis of factors being associated with a poor outcome:	
			▪ motor vehicle crashes (*p* < 0.001) ▪ non-accidental trauma injury (*p* = 0.002) ▪ injuries that occurred outside home (*p* = 0.007)	
Feng et al. 2015 [20]	261 Children aged 16 years or less. Admitted for injuries sustained as pedestrians.	ED of a tertiary children’s hospital.	Gender	◆ Correct possible parental misconception allowing child to be unaccompanied/unsupervised pedestrians. ◆ Encourage proper supervision of children especially while travelling on the roads.
			▪ Male: 60.5% ▪ Female: 39.5%	
			Age group	
			▪ Aged 1–3 years: 7.3% ▪ Aged 4–6 years: 16.9% ▪ Aged 7–12 years: 50.2% ▪ Aged 13–16 years: 25.7%	
			Site of accident	
			▪ Roadway: 81.2% ▪ Car park: 8.4% ▪ Sidewalk: 6.5%	
			67.8% of all subjects were unaccompanied by an adult.	
			Factors associated with major trauma	
			▪ Positive history of being flung (*p* = 0.001) ▪ Loss of consciousness (*p* < 0.001)	
Ngo et al. 2005 [21]	353 Children aged less than 16 years presenting with suspected foreign body (FB) in the ear, nose or throat.	ED of a tertiary children’s hospital.	Age group (in years)	◆ Recognising the epidemiological profile of children presenting with FB in the ear, nose or throat. ◆ ED physician able to manage most FB cases.
			▪ <4: 39.4% ▪ 4 to <8: 43.3% ▪ 8 to <12: 12.7% ▪ 12 to <16: 4.5%	
			Ear FB	
			▪ Majority (43.3%) aged between 4 and 8 years old. ▪ Commonest object: toy parts. ▪ Commonest presentation: Local pain (47%)	
			Throat FB	
			▪ Most common object: fish bone (81.1%). ▪ Most common presentation: Local pain (89.2%)	
			Nasal FB	
			▪ Commonest objects: beads, toy parts and organic matter.	
Ho et al. 1998 [22]	112 Children admitted to the paediatric ward for accidental poisoning.	A paediatric ward in a tertiary hospital.	Demographics	◆ Store drugs in properly secured and locked cupboards. ◆ Do not use beverage bottles for storing of toxic liquids. ◆ Child-proof containers and child resistant packaging. ◆ Health education to children and caregivers.
			▪ Males: 54% ▪ 60% were aged between 1 and 3 years.	
			Type of ingestion	
			▪ Medications: 49% ▪ Household liquids: 16%	
			Mean hospital stay: 2.4 days	
			Most common reason for unsupervised child: Caregiver pre-occupied with housework (23%)	
			Majority of the medications was	
			▪ Kept in unlocked cupboards ▪ Placed on tables, refrigerators, or left in bags either before or after consumption.	
**Childhood Injuries related to Specific Devices or Accessories**				
Tripathi et al. 2016 [23]	248 Children aged less than 6 years. Pram or stroller related injuries.	Injury surveillance database of a tertiary children’s hospital ED.	Median age: 12.5 months.	◆ Appropriate selection of prams and strollers according to age and size of child, to prevent injury. ◆ Ensure proper use of device and supervision of child at all times. ◆ Safety checks with appropriate adjustments prior to usage. ◆ Mandatory safety features. ◆ Special labels and cues sited near injury prone locations. ◆ Installation of barricades at escalator entrances and exits.
			Type of injury	
			▪ Blunt injuries: 97.6% ▪ Crushing injury: 2.4%	
			Location of injury	
			▪ Home injuries: 46.8% ▪ Outside home injuries: 52.8% ▪ At shopping malls: 8.4%	
			Mechanism of injury	
			▪ Fall/tripping: 89.1% ▪ Hit against pram parts: 6.5% ▪ Entrapment injuries: 3.2% ▪ Others: 1.2%	
			20.1% required procedural intervention.	
			17.7% admitted for head injury.	
			1.6% admitted for procedure under LA.	
			Injuries requiring procedural intervention were significantly associated with older age (*p* < 0.001) compared with children who were younger.	
			Entrapment injuries were significantly associated with the indication for intervention within the ED (*p* < 0.001) compared with other mechanisms of injury.	
Thein et al. 1997 [24]	185 Parents or caregivers of infants aged 7 to 10 months attending developmental assessment.	A polyclinic (primary care centre).	90% used walkers regularly.	◆ Avoid the use of infant walkers. ◆ Consider safer alternative of a crib or playpen. ◆ Health education to parents of the possible hazards of using infant walkers.
			Walker-using group	
			▪ 12.5% had one or more injuries. ▪ Longer duration of usage associated with higher incidence of injuries (*p* = 0.43). ▪ DDST-S results: 7.2% abnormal, 3.6% questionable.	
			Group not using walker	
			▪ DDST-S results: 100% normal.	
**Playground-Related Injuries**				
Tan et al. 2007 [25]	19,094 Children up to 16 years of age with unintentional injuries.	3 ED, 2 polyclinics (primary care centres), HSA.	Incidence of play-ground related injuries: 8.5%	◆ Need for a review of playground equipment’s height. ◆ Replace monkey bars with alternative safer equipment. ◆ Providing safety advice at playgrounds to educate children and caregivers on proper use of play-ground equipment. ◆ Proper profiling records for injury prevention initiatives.
			Increasing male predominance with increasing age groups (*p* = 0.006)	
			Mechanism of injury	
			▪ Falls: 70.7% ▪ Contact with blunt objects: 12.6% ▪ Application of bodily force: 4.1% ▪ Crushing injuries: 0.8%	
			Major sites of injury	
			▪ Upper limb fractures: 18.2% ▪ Lower limb fractures: 3.0% ▪ Head injuries: 4.3%	
			Commonest causative playground equipment	
			▪ Monkey bar or other playground climbing apparatus: (52.1%) ▪ Slide: 21.2% ▪ Swing: 6.3%	
			Risk of sustaining an upper limb fracture at a playground was highest amongst children who were between 6 to 10 years of age compared to the other age groups (*p* < 0.001).	
			Falls arising from more than 1m in height had a 4.1 times higher risk of an injury involving upper limb fractures (OR = 4.1, *p* < 0.001) compared to falls from less than 1 m in height.	
			Children who were not accompanied by any caregivers were 1.4 times more likely to sustain upper limb fractures (*p* < 0.05) compared to children whom are accompanied by caregivers.	
Mahadev et al. 2004 [26]	390 Children with playground-related extremity fractures.	A tertiary children’s hospital	Male:Female ratio = 2:1	◆ Need for a safer playground environment. ◆ Replace monkey bars and hanging equipment. ◆ Limit height limit of playground equipment to 1500 mm or less.
			Mean age: 7 years	
			Contribution of the type of equipment to extremity fractures.	
			▪ Monkey Bar: 66% ▪ See Saw 15% ▪ Slide 10% ▪ Swing 8% ▪ Flying fox: 1%	
			Location of injury	
			▪ Upper extremity: 92.8% ▪ Lower extremity: 7.2%	
Leung et al. 2011 [27]	226 All playground related extremity fractures.	A tertiary children’s hospital.	Mean age: 7.5 years.	◆ Reducing playground injury will translate to significant financial savings as well as reduced psychosocial effects.
			Ratio between male:female = 2:1	
			Location of injury	
			▪ Public areas: 92.3% ▪ Schools: 5% ▪ Private locations: 2.7%	
			Site of injury	
			▪ Forearm fractures: 36% ▪ Supracondylar fracture: 29%	
			Contribution of the type of equipment to extremity fractures:	
			▪ Monkey bars: 49% ▪ Slides: 14%	
			Type of treatment rendered	
			▪ Casting: 65% ▪ Closed manipulation and reduction: 24% ▪ Surgery: 11%	
			1.2% of all cases required admission.	
			Cost involved: $247.50–$3792.50 per patient.	
Lam et al. 2013 [28]	267 Children less than 17 years old with playground-related fractures.	A tertiary children’s hospital.	Mean age: 7 years.	◆ Monkey bars should be replaced with safer alternative equipment. ◆ Ensure proper supervision of the child at playgrounds. ◆ Maintaining the child’s BMI within the recommended limits may reduce the incidence of severe fractures.
			Incidence of upper limb fractures: 95.5%	
			Contribution of the type of equipment to extremity fractures	
			▪ Monkey bars: 45.7% ▪ Slides: 14.6%	
			The presence of any supervision significantly correlate with a lower incidence of major fracture compared to injuries sustained in the absence of any supervision (*p* = 0.000).	
			In those group of injuries sustained in the presence of supervision, it was found that supervision from parents (18.8%) (*p* = 0.004) or siblings (16.7%) resulted in a lower incidence of major fractures, compared to grandparents (27.3%) or domestic helpers (25.9%).	
			Increased incidence in major fractures occurring in children with a Body-Mass Index (BMI) at either ends of the extreme (10 percentile or less and 95 percentile or more) (37.9%) compared to those with a BMI between 11 to 94 percentile (27.3%) (*p* = 0.074).	
**Injuries Related to Drowning**				
Tyebally et al. 2010 [29]	38 All children seen for drowning and near-drowning.	ED of Singapore Health Services network; HSA	Median age: 6.3 years	◆ Ensure proper supervision of children near water hazards. ◆ Importance of having adequate safety features such as fencing around pools, clear demarcation of seaside swimming areas. ◆ Mandatory presence of lifeguard on duty at specific time. ◆ Discourage building of water features which may have hazardous features.
			Males: 57.9%	
			Mortality rate: 23.7%	
			Major locations where injury was sustained	
			▪ Swimming pool: 52.6% ▪ Sea: 21.1% ▪ Pond: 7.9%	
			In 47.4% of the cases, there was no safety features at location of the injury.	
			Only 23.6% of the injuries were witnessed by caregivers;	
			100% of the near drowning cases at swimming pool survived when lifeguard was present.	

ED: Emergency Department; BMI: Body-Mass Index; HSA: Department of Forensic Medicine, Health Science Authority; DDST-S: Singapore modified version of the Denver Developmental Screening Test.

**Table 2:** Proposed Recommendations in prevention of Childhood injuries in Singapore based on Strength of Recommendation Taxonomy (SORT).

Proposed Recommendation		Evidence Rating
Home injuries		
1.	Raising awareness and reducing common home hazards such as furniture hazards, dangling electrical cord, exposed sharp objects [15,19,30].	C
2.	Providing education and interventional programs on first aid and fall prevention strategies [16,17].	C
3.	Dissuading local families from nursing infants or children in adult beds and to encourage the use of proper cots [18].	C
4.	Increasing the usage of safety gates, non-slip mats, door-stopper or self-closing hinges [18,41].	C
5.	Regular education to both caregivers and children on poison prevention and proper storage of drugs and chemicals at home (with proper labels and securely locked cupboards) [22,32].	C
6.	Manufacturing of drugs in child-proof containers, opaque blister packs or strips [22].	C
Childcare products and footwear		
Prams and Strollers		
7.	Safety checks with appropriate adjustments to ensure a stable and well-balanced structure and absence of any exposed joints or hinges prior to each use [23].	C
8.	Providing constant supervision of children and ensuring the use of safety harness [23].	C
9.	Ensuring presence of mandatory safety features which are in compliance with international safety standards or certifications [23].	C
10.	Special labels and cues sited near injury prone locations such as stairways and escalators; installation of barricades at their exits [23].	C
Escalator Safety and use of rubber clogs		
11.	Essential safety features on escalators with regular maintenance and lubrication of side panels [33].	C
12.	Supervision and accompaniment of children by an adult; disallow playing while on escalators [33].	C
13.	To be mindful of the possibility of any clothing with strings or straps that may be trapped while travelling [33].	C
14.	Ensuring safe distance between child and sides of escalator; hold onto handrail and face forward [33].	C
Heelys™, infant walkers, toys, high chairs		
15.	Wearing of safety gear and close supervision by an adult when using Heelys™ [34].	C
16.	Avoiding the use of infant walkers; consider safer alternative of a crib or playpen [17,24].	C
17.	Routine inspection of toys for potential hazards; replace or repair damaged toys immediately [35].	C
18.	Adhere to the recommended age group of each toy in their selection for their children [35].	C
19.	Ensure fastening of seatbelts with the use of high chairs [9].	C
Sarong cradles		
20.	The dangers and potential morbidity in the use of sarong cradles should be publicized and have its usage discouraged [17,18,35].	C
21.	Dedicated supervision by a responsible adult; ensuring the usage of proper sarong length; routine inspections for potential defects; usage of an appropriate protective material around the area of the cradle, to reduce sarong-related injuries [35].	C
Playground injuries		
22.	Review of the heights of common playground equipment to ensure that they are age-appropriate [25].	C
23.	Restricting the maximum height of any hanging equipment to 1500 mm [18,26].	C
24.	Prominent displays of safety advice on the use of equipment in the playground [25].	C
25.	Monkey bars should be substituted with safer equipment [18,25,26,27].	C
26.	Maintaining the child’s BMI within the recommended limits [28].	C
Transportation-related injuries		
27.	More publicity efforts to boost the awareness on the updated legislation in Singapore with regards to the obligatory usage of appropriate child restraints or booster seats in cars for anyone below the height of 1.35 m [16,17,18,19,42].	C
28.	Stricter enforcement of the child restraint seat law [18].	C
29.	Introduction of child safety programs at the ED [19].	C
30.	Mandatory use of helmets while riding bicycles at all times; wearing of proper footwear for cycling [18,43].	C
31.	Not allowing young children to manoeuvre roads or car parks alone but to ensure that they are properly supervised [20].	C
Drowning and near drowning		
32.	Avoid having water-filled pails at home or to ensure that children do not have access to these pails or ponds at home [18,29,30].	C
33.	Adult supervision should always be present when bathing infants or toddlers [29]; and when children are at swimming pools, seas or any water features [29,37].	C
34.	Having adequate essential safety features at swimming pools and sea-side [29,37].	C
35.	Considering formal legislatures of the need for lifeguards and pool fencing [18,29,37].	C
36.	Acquiring of swimming skills and access to aquatic safety education among all children [37].	C
Role of healthcare professionals and nation-wide initiatives		
37.	Advocates of health education and provision of injury prevention advice during antenatal or postnatal visits, as well as immunization and developmental assessment sessions [16,17].	C
38.	Education of caregiver on fall prevention strategies to increase their awareness and preventing inpatient falls in children [44].	C
39.	Establishment of a robust injury surveillance database for epidemiological analysis and enabling targeted future initiatives for safety campaigns and injury prevention [18,25].	C

Evidence rating A: Recommendation based on consistent and good-quality patient-orientated evidence [45]; Evidence rating B: Recommendation based on inconsistent or limited-quality patient-orientated evidence [45]; Evidence rating C: Recommendation based on consensus, usual practice, opinion, disease-orientated evidence, or case series for studies of diagnosis, treatment, prevention or screening [45].
